# Disrupted principal network organisation in multiple sclerosis relates to disability

**DOI:** 10.1038/s41598-020-60611-4

**Published:** 2020-02-27

**Authors:** Thalis Charalambous, Jonathan D. Clayden, Elizabeth Powell, Ferran Prados, Carmen Tur, Baris Kanber, Declan Chard, Sebastien Ourselin, Claudia A. M. Gandini Wheeler-Kingshott, Alan J. Thompson, Ahmed T. Toosy

**Affiliations:** 1 Queen Square MS Centre, Department of Neuroinflammation, UCL Institute of Neurology, Faculty of Brain Sciences, University College London, London, UK; 2 UCL GOS Institute of Child Health, University College London, London, UK; 3 Medical Physics and Biomedical Engineering, University College London, London, UK; 4 Center for Medical Imaging Computing, Medical Physics and Biomedical Engineering, UCL, London, WC1V 6LJ UK; 5 eHealth Centre, Universitat Oberta de Catalunya, Barcelona, Spain; 6 Brain MRI 3T Research Center, C. Mondino National Neurological Institute, Pavia, Italy; 7 Department of Brain and Behavioural Sciences, University of Pavia, Pavia, Italy

**Keywords:** Diagnostic markers, Multiple sclerosis

## Abstract

Structural network-based approaches can assess white matter connections revealing topological alterations in multiple sclerosis (MS). However, principal network (PN) organisation and its clinical relevance in MS has not been explored yet. Here, structural networks were reconstructed from diffusion data in 58 relapsing-remitting MS (RRMS), 28 primary progressive MS (PPMS), 36 secondary progressive (SPMS) and 51 healthy controls (HCs). Network hubs’ strengths were compared with HCs. Then, PN analysis was performed in each clinical subtype. Regression analysis was applied to investigate the associations between nodal strength derived from the first and second PNs (PN1 and PN2) in MS, with clinical disability. Compared with HCs, MS patients had preserved hub number, but some hubs exhibited reduced strength. PN1 comprised 10 hubs in HCs, RRMS and PPMS but did not include the right thalamus in SPMS. PN2 comprised 10 hub regions with intra-hemispheric connections in HCs. In MS, this subnetwork did not include the right putamen whilst in SPMS the right thalamus was also not included. Decreased nodal strength of the right thalamus and putamen from the PNs correlated strongly with higher clinical disability. These PN analyses suggest distinct patterns of disruptions in MS subtypes which are clinically relevant

## Introduction

Multiple sclerosis (MS) is an inflammatory, demyelinating and neurodegenerative disease of the central nervous system (CNS)^1^. Conventional whole brain magnetic resonance imaging (MRI) measures do not necessarily reflect processes of brain reorganisation in pathology and poorly reflect the long-term course of the disease^2^ meaning that additional biomarkers for disease progression and treatment effects are needed.

Structural network analysis provides a framework to study whole brain connectivity patterns and their disruptions, incorporating data beyond focal pathology (i.e. lesions). In this approach, grey matter regions are modelled as nodes connected by structural pathways known as edges derived from diffusion data. The pairwise connection between nodes can be represented in a connectivity matrix and graph theory is applied to quantify differences in connectivity patterns in pathology^3^.

The application of network-based approaches in MS has shown interesting findings. Previous studies demonstrated that network measures were different between MS patients and controls^4–6^ or between clinical profiles^7,8^. Additionally, structural network measures were associated with clinical disability^9^ and lesion load^6^ and with cognitive deficits^4^. Interestingly, structural network metrics explained physical disability and cognitive impairment over and above non-network measures^8^ highlighting the clinical relevance of these studies in MS.

Network-based techniques have the potential to not only allow the quantitative characterisation of global connectivity patterns but also to provide a framework to elucidate important topological features. For instance, studies have identified the existence of a number of highly connected regions, hubs, and how these are affected in MS^6^. It has also been proposed that hub nodes have the tendency to be more densely connected with each other^10^ and in fact such ‘rich-club’ organisation is affected in MS^11,12^. Despite these promising results, further work is needed to gain deeper understanding of the topological alterations occurring in MS.

Recently, a data-driven framework for structural network decomposition has been proposed which provides stable, meaningful and reproducible subnetworks with strong internal connectivity^13^. Briefly, applying the principal component analysis-based technique, the full connectivity matrix is decomposed into partial connectivity matrices through linear decomposition. The derived subnetworks, namely principal networks (PNs) are ranked based on their internal connectivity such that the first PN (PN1) is the most interconnected subnetwork^13^. Decomposing the whole network into subnetworks may reveal changes otherwise undetected and understanding how these are affected in pathology could provide potential biomarkers to monitor disease progression and treatment effects. Applications of PNs in MS are yet to be explored.

The aim of this study was (a) to characterise the PNs in MS and (b) to explore their relationships with motor disability and information processing speed impairment in a previously studied cohort^8^.

## Methods

### Participants

This study included 122 MS patients (58 RRMS (40 female, mean age (±SD) 49 ± 12 years), 28 PPMS (18 female, mean age 46 ± 9 years) and 36 SPMS (28 female, mean age 52 ± 9 years)) and classified based on Lublin and Reingold criteria^14^. Fifty-one HCs (26 female, mean age 41 ± 13 years) not known to have neurological or psychiatric disorder were also examined. Participants underwent MRI assessment and neurological assessment using EDSS (Expanded Disability Status Scale)^15^. Information processing speed capacity was also assessed using SDMT (Symbol Digit Modalities Test) in a subset of MS patients (n = 60; eTable 1 Supplemental results). Additional information for these patients can be found in the supplemental material. Written informed consent was obtained for participation in the study, which was approved by the Institutional Ethics Committee of the National Hospital of Neurology and Neurosurgery, University College London Hospital NHS foundation trust. All methods were performed in accordance with the relevant guidelines and regulations. Demographics of the participants are summarised in Table 1.

**Table 1:** Demographics Of The Study Participants.

	HCs (n = 51)	MS patients (n = 122)	RRMS (n = 58)	PPMS (n = 28)	SPMS (n = 36)	*P values
**Demographics**						
Age, years	41 ± 13	48 ± 11	42 ± 10	52 ± 9	53 ± 7	**p** < **0**.**001**^**a**^
Gender (M/F)	25/26	36/86	18/40	10/18	8/28	p = 0.06^b^
Disease duration, years	—	15 ± 10	11 ± 8	14 ± 7	22 ± 10	**p** < **0**.**001**^**a**^
% (no) patients of DMTs	—	58 (67)	84 (48)	13 (3)	47(16)	**p** < **0**.**001**^**b**^
LL (ml)	—	14.37 ± 15.92	12.78 ± 15.72	16.56 ± 19.83	15.23 ± 12.73	**p** < **0**.**001**^**a**^
**Clinical scores**						
EDSS, median	—	5.5 (0–8.5)	2 (0–7)	6 (3–8)	6.5 (4–8.5)	**p** < **0**.**001**^**a**^
SDMT	65.08 ± 8.31	45.50 ± 13.27	51.04 ± 14.28	42.86 ± 9.46	39.00 ± 10.88	**p** < **0**.**001**^**a**^

Abbreviations: HCs = Healthy controls; MS = multiple sclerosis; RRMS = relapsing remitting MS; PPMS = primary progressive MS; SPMS = secondary progressive MS; DMT = Disease Modifying Treatment; LL = Lesion load; EDSS = Expanded Disability Status Scale; SDMT = Symbol Digit Modalities Test; ANOVA = analysis of variance.

^*^P values in bold denote statistical significance at p < 0.05; statistical test between the various clinical subtypes and healthy controls where applicable.

^a^One-way ANOVA.

^b^Chi Squared test.

### MRI data acquisition

MRI data were acquired using a Philips Achieva 3 T MR scanner (Philips Healthcare, Best, Netherlands) using a 32-channel coil. The high angular resolution diffusion imaging (HARDI) scan consisted of a cardiac-gated spin-echo (SE) sequence with echo planar imaging (EPI) readout (resolution = 2 × 2 × 2 mm^3^, repetition time (TR) = 24000 ms; echo time (TE) = 68 ms; 61 isotropically distributed diffusion-weighted directions, b-value = 1200 s/mm^2^, 7 b = 0 volumes, field of view 112 × 112, number of slices 72). In each subject, the following data were also acquired: (1) T1-weighted images acquired using a 3D fast-field echo scan (resolution = 1 × 1 × 1 mm^3^, TR = 6.9 ms, TE = 3.1 ms, inversion time (TI) = 824.5 ms) and (2) dual-echo proton density/T2-weighted axial oblique scans (resolution = 1 × 1 × 3 mm^3^, TR = 3500 ms, TE = 19/85 ms, field of view 240 × 180, number of slices 50). All data were acquired with slices aligned with the anterior commissure (AC) – posterior commissure (PC) line to minimise the effect of head positioning on data analysis.

### Structural imaging processing

Subsequent steps for network reconstruction are reported elsewhere^8^. Briefly, a non-rigid transformation was performed to register the subjects’ non-filled T1-weighted bias-field corrected image to the corresponding diffusion-weighting image (DWI) using BrainSuite v.15b^16^. The target volume was the first b = 0 image after DWI pre-processing, resulting in a structural image of resolution 2 × 2 × 2 mm^3^. T2-hyperintense lesions were manually delineated from the PD-T2-weighted scans using JIM (v6.0, Xinapse Systems, Aldwincle, UK), non-rigidly transformed to DWI space and then filled the T1-weighted images using a modality-agnostic patch-based method. The filled T1-weighted images were then segmented into different tissue types and parcellated according to Desikan–Killiany–Tourville atlas protocol using GIF^17,18^.

### Diffusion-weighted imaging processing and tractogram reconstruction

The mean b0 obtained from the extra 7 b0s was registered to the first b0 of the diffusion data. Then, using the obtained transformation, we resampled the extra b0s to the DWI space. We then corrected for eddy current, head motion^19^ and EPI distortions^16^. We used second order integration over fibre orientation distributions (iFOD2) estimated with constrained spherical deconvolution (CSD)^20^ for probabilistic tractography. 10^7^ streamlines were generated by implementing the anatomically constrained tractography (ACT) algorithm^21^ followed by spherical-deconvolution informed filtering of tractograms (SIFT2)^22^. Additionally, by providing a white matter mask during tractogram reconstruction, as part of the ACT step, we ensured that no streamlines were incorrectly terminated in white matter due to the presence of lesions^8^.

### Structural network and principal networks reconstruction

For the structural brain network, we defined as nodes the cortical and subcortical regions, and as edges the sum of the weights of streamlines connecting a pair of nodes resulting in a symmetrical network of 115 nodes^22^. We generated one brain network per subject, estimated the mean connectivity matrix for each group and then defined hubs as regions that exhibited higher strength (≥ mean + 1 standard deviation (SD))^23^. Strength is defined as the sum of all edge weights connected to the node.

For PN estimation, we applied PN analysis to the mean connectivity matrix for each clinical profile using the default loading threshold of 0.1^13^. Loadings are the normalised eigenvectors of the connectivity matrix. Their magnitude corresponds to the influence of each node to the PN and the sign reflects the sense of their connectivity, relative to the other nodes. This decomposition method is implemented in TractoR (http://www.tractor-mri.org.uk)^24^. The derived subnetworks are ranked based on their internal connectivity such that PN1 is the most strongly interconnected subnetwork. We focused on PN1 and PN2 as they have the highest and second highest internal connectivity.

To estimate PN specific measures, we used PN1 and PN2 defined in HCs as a basis to estimate the connectivity matrices for the HCs and MS subtypes. Effectively, we constructed two symmetrical subnetworks with 10 nodes each and then estimated the nodal strengths in each subnetwork.

### Statistical analysis

Statistical analysis was performed using R software (https://www.r-project.org/ v3.3.0). To explore nodal strength differences between MS patients and HCs we used regression analysis adjusting for age, gender, lesion load, disease duration and total intracranial volume (TIV). To evaluate whether the derived PNs are clinically relevant we performed a post-hoc analysis on the subnetwork nodal strengths of right putamen and right thalamus as they tended to be lost from PN1 and PN2 in MS subtypes. Specifically, multiple regression analyses were performed in the whole MS population, in which clinical scores (EDSS or SDMT) were considered, in turn, as the dependent variable. As independent variables we included the PN1 or PN2 nodal strengths (separately, in different models) together with age, gender, disease duration and lesion load. Because the right putamen and thalamus were subcortical regions, deep grey matter volume was also added to the model as a covariate. P-values < 0.05 were considered statistically significant. For completion, we also performed an exploratory analysis by constructing a pairwise univariate association matrix that includes all brain regions belonging to PN1 and PN2 and the clinical scores.

## Results

### Network hubs analysis

Table 2 shows the identified network hubs for HCs, RRMS, PPMS and SPMS. There were 18 network hubs in HCs and MS subtypes indicating preserved hub nodes and number across all groups under investigation. We detected significantly reduced strength in some hubs in MS subtypes. The number of regions with reduced strength increased in more progressive phenotypes. For instance, compared to HCs, in RRMS, 6 hub regions showed decreased strength whereas in SPMS 13 out of 18 regions exhibited statistically reduced strength. Also, the deep nuclei showed reduced strength in MS subtypes compared to HCs **(**Table 2; p < 0.05). All models were adjusted for age, gender, lesion load, disease duration and TIV.

**Table 2:** Network Hubs In Healthy Controls And Multiple Sclerosis Subtypes.

Brain region	HCs (x10^5^)	RRMS (x10^5^)	PPMS (x10^5^)	SPMS (x10^5^)
**Frontal lobe**				
*Right precentral gyrus*	3.39 (0.21)	3.34 (0.27)	3.27 (0.24)	3.23 (0.23)
*Left precentral gyrus*	3.53 (0.23)	**3**.**38 (0**.**28)**	**3**.**36 (0**.**30)**	**3**.**34 (0**.**32)**
*Right superior frontal gyrus*	3.37 (0.18)	**3**.**22 (0**.**23)**	**3**.**11 (0**.**26)**	**3**.**09 (0**.**25)**
*Left superior frontal gyrus*	3.28 (0.17)	3.20 (0.26)	3.10 (0.03)	3.13 (0.24)
*Right middle frontal gyrus*	2.88 (0.20)	2.78 (0.25)	2.75 (0.23)	**2**.**58 (0**.**22)**
*Left middle frontal gyrus*	3.04 (0.19)	2.92 (0.27)	**2**.**77 (0**.**23)**	**2**.**76 (0**.**22)**
**Parietal lobe**				
*Right postcentral gyrus*	2.25 (0.10)	2.28 (0.16)	2.20 (0.16)	2.16 (0.18)
*Left postcentral gyrus*	2.32 (0.12)	2.27 (0.14)	2.31 (0.19)	2.32 (0.18)
Right superior parietal lobule	2.26 (0.10)	2.21 (0.13)	**2**.**09 (0**.**16)**	**2**.**08 (0**.**15)**
Left superior parietal lobule	2.01 (0.10)	1.96 (0.12)	1.93 (0.13)	**1**.**85 (0**.**11)**
Right precuneus	2.20 (0.10)	2.14 (0.13)	2.10 (0.16)	**1**.**98 (0**.**13)**
Left precuneus	2.01 (0.10)	1.95 (0.12)	1.94 (0.13)	**1**.**85 (0**.**12)**
**Temporal lobe**				
Right middle temporal gyrus	2.06 (0.10)	1.96 (0.13)	**1**.**89 (0**.**12)**	**1**.**88 (0**.**12)**
Left middle temporal gyrus	1.85 (0.09)	1.78 (0.12)	1.73 (0.10)	1.74 (0.11)
**Subcortical grey matter**				
*Right thalamus*	2.67 (0.15)	**2**.**32 (0**.**02)**	**2**.**27 (0**.**19)**	**2**.**08 (0**.**15)**
*Left thalamus*	2.53 (0.15)	**2**.**22 (0**.**13)**	**2**.**23 (0**.**17)**	**2**.**06 (0**.**14)**
Right putamen	2.34 (0.13)	**2**.**13 (0**.**12)**	**2**.**11 (0**.**02)**	**2**.**03 (0**.**13)**
Left putamen	2.20 (0.09)	**2**.**03 (0**.**11)**	**2**.**04 (0**.**15)**	**1**.**94 (0**.**13)**

Average values and standard deviations (SD) of region-specific strength.

Bold values represent regions that exhibit significant decrease in nodal strength against HCs. The italicized regions are those that belong to the first principal network in healthy controls.

Abbreviations: HCs = healthy controls; RRMS = relapsing-remitting MS; PPMS = primary progressive MS; SPMS = secondary progressive MS.

Reproduced from Charalambous^44^.

### Principal network analysis

**Figure 1:**
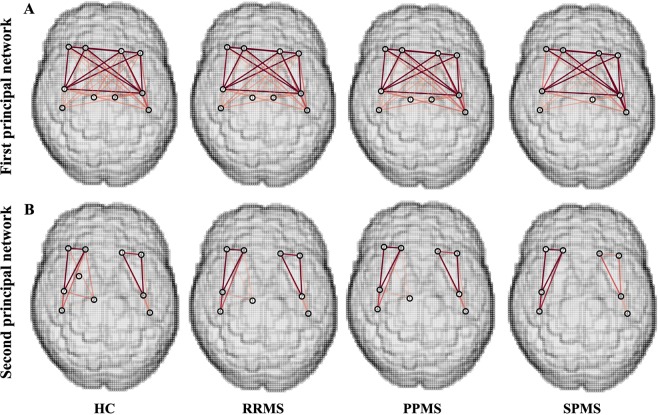
The first and second principal network in healthy controls and multiple sclerosis subtypes. (**A**) There is a loss of the right thalamus in the first principal network in SPMS. (**B**) For the second principal network, there is a loss of the right putamen connections in all MS subtypes, and an additional loss of the right thalamus in SPMS. Intensity of the edges’ colour denotes the strength of the connection. Abbreviations: HCs = healthy controls; RRMS = relapsing-remitting MS; PPMS = primary progressive MS; SPMS = secondary progressive MS. Reproduced from Charalambous^44^.

Figure 1A and Table 3 demonstrate PN1 identified in HCs consisted of 10 regions that are a subset of the previously defined hubs Table 2. Figure 1A shows that the connectivity of PN1 predominantly includes inter-hemispheric connections with important intra-hemispheric connections. We used the same loading threshold (0.1) to identify PN1 in each MS group. In RRMS and PPMS, PN1 is the same as in HCs, whereas in SPMS a loss of the right thalamus was detected (Fig. 1A, Table 3 and eFig. e1 Supplemental Results).

**Table 3:** First Principal Network Nodes In Healthy Controls And Multiple Sclerosis Subtypes.

1^st^ principal network nodes			
HCs	RRMS	PPMS	SPMS
**Frontal lobe**			
Right precentral gyrus	Right precentral gyrus	Right precentral gyrus	Right precentral gyrus
Left precentral gyrus	Left precentral gyrus	Left precentral gyrus	Left precentral gyrus
Right superior frontal gyrus	Right superior frontal gyrus	Right superior frontal gyrus	Right superior frontal gyrus
Left superior frontal gyrus	Left superior frontal gyrus	Left superior frontal gyrus	Left superior frontal gyrus
Right middle frontal gyrus	Right middle frontal gyrus	Right middle frontal gyrus	Right middle frontal gyrus
Left middle frontal gyrus	Left middle frontal gyrus	Left middle frontal gyrus	Left middle frontal gyrus
**Parietal lobe**			
Right postcentral gyrus	Right postcentral gyrus	Right postcentral gyrus	Right postcentral gyrus
Left postcentral gyrus	Left postcentral gyrus	Left postcentral gyrus	Left postcentral gyrus
**Subcortical grey matter**			
Right Thalamus Proper	Right Thalamus Proper	Right Thalamus Proper	
Left Thalamus Proper	Left Thalamus Proper	Left Thalamus Proper	Left Thalamus Proper

Abbreviations: HCs = healthy controls; RRMS = relapsing-remitting MS; PPMS = primary progressive MS; SPMS = secondary progressive MS.

Reproduced from Charalambous^44^.

Figure 1B and Table 4 show that PN2 in HCs is comprised of 10 brain regions that are very similar to PN1 with the exception of the right putamen and left thalamus. Specifically, the right putamen is identified as network hub in PN2 while the left thalamus no longer qualifies. Additionally, PN2 is comprised of regions with strong intra-hemispheric connections **(**Fig. 1B**)**. In all MS subtypes, this network did not include the right putamen compared to HCs, while in SPMS group there was additional loss of the right thalamus (Fig. 1B and eFig. 2 Supplemental Results).

**Table 4:** Second Principal Network Nodes In Healthy Controls And Multiple Sclerosis Subtypes.

2^nd^ principal network nodes			
HCs	RRMS	PPMS	SPMS
**Frontal lobe**			
Right precentral gyrus	Right precentral gyrus	Right precentral gyrus	Right precentral gyrus
Left precentral gyrus	Left precentral gyrus	Left precentral gyrus	Left precentral gyrus
Right superior frontal gyrus	Right superior frontal gyrus	Right superior frontal gyrus	Right superior frontal gyrus
Left superior frontal gyrus	Left superior frontal gyrus	Left superior frontal gyrus	Left superior frontal gyrus
Right middle frontal gyrus	Right middle frontal gyrus	Right middle frontal gyrus	Right middle frontal gyrus
Left middle frontal gyrus	Left middle frontal gyrus	Left middle frontal gyrus	Left middle frontal gyrus
**Parietal lobe**			
Right postcentral gyrus	Right postcentral gyrus	Right postcentral gyrus	Right postcentral gyrus
Left postcentral gyrus	Left postcentral gyrus	Left postcentral gyrus	Left postcentral gyrus
**Subcortical grey matter**			
Right Thalamus Proper	Right Thalamus Proper	Right Thalamus Proper	
Right Putamen			

Abbreviations: HCs = Healthy controls; RRMS = relapsing-remitting MS; PPMS = primary progressive MS; SPMS = secondary progressive MS;

Reproduced from Charalambous^44^ with permission.

### Associations between nodal principal network strength and clinical scores in multiple sclerosis patients

We found that lower strengths of the right thalamus in PN1 (p = 0.014), of the right thalamus in PN2 (p = 0.007) and of the right putamen in PN2 (p = 0.006) were associated with higher EDSS score across all MS patients **(**Table 5**)**. Additionally, lower strengths of the right thalamus (p = 0.002) and of the right putamen (p = 0.002), both in PN2, were associated with worse (lower) SDMT scores in all patients. There was borderline evidence of an association between lower nodal PN1 strength of the right thalamus with lower SDMT (p = 0.081) also in all patients. All models were adjusted for age, gender, lesion load, disease duration and deep grey matter volume.

**Table 5:** Multiple Regression Analysis Between Nodal Strengths And Clinical Scores in Multiple Sclerosis patients.

PN1 nodal strength	Regression coefficient	Confidence intervals	P value	PN2 nodal strength	Regression coefficient		Confidence intervals	P value	
**EDSS**									
**Right Thalamus**	−2.92 × 10^−5^	−5.25 × 10^−5^ to −0.59 × 10^−5^	**0**.**014**	**Right Thalamus**		−2.76 × 10^−5^	−4.77 × 10^−5^ to −0.74×10^−5^		**0**.**007**
				**Right Putamen**		−4.68 × 10^−5^	−8.02 × 10^−5^ to −1.35 × 10^−5^		**0**.**006**
**SDMT**									
**Right Thalamus**	1.78 × 10^−3^	−2.27 × 10^−5^ to 3.79 × 10^−3^	0.081	**Right Thalamus**		2.88 × 10^−4^	1.12 × 10^−4^ to 4.64 × 10^−4^		**0**.**002**
				**Right Putamen**		5.05 × 10^−4^	1.92 × 10^−4^ to 8.19 × 10^−4^		**0**.**002**

P values denote statistical significance at p < 0.05.

PN = principal network; EDSS = Expanded Disability Status Scale; SDMT = Symbol Digit Modalities Test.

Additional post-hoc exploratory analysis that included all brain regions belong to the PN1 and PN2 showed that the higher correlation coefficient between clinical scores disability and brain regions is shown in PN1 right thalamus, PN2 right thalamus and PN2 right putamen. (eFig. 3; Supplemental Results**)**.

## Discussion

In this study, we first characterised the well-studied network hubs, then evaluated the subnetwork topological changes that occur in MS subtypes through PN analysis and whether these changes are clinically relevant. This technique decomposes the whole network into subnetworks and ranks them based on their internal connectivity^13^. Here, we focused on PN1 and PN2 as these subnetworks include the most interconnected nodes. The study findings show that compared to controls, MS patients had preserved hubs in terms of regions and quantities, but reduced strength in some hubs. When we studied the subnetworks, we found that deep grey matter connections are affected in MS and their nodal strengths are associated with EDSS and SDMT independently.

### Structural network hubs

Within the framework of network science, nodes that are found to have a central role in the network are generally referred to as network hubs. In this study, we identified 18 hub nodes, including regions from frontal, temporal and parietal lobes and deep grey matter structures. Previous studies using the same tractography reconstruction methods showed high overlap of nodes identified as hubs^25^ despite the usage of different parcellation schemes (i.e. Desikan-Killiany vs GIF^17,26^).

The relevance of network hubs has already been studied in MS. Some nodes exhibited increased strength and were hence classified as hubs in MS and not in controls^4,6^, whilst others showed preserved hub distribution in RRMS compared to HCs^27^. Here, we report relatively preserved hub node and number not only in RRMS but also in PPMS and SPMS. When comparing the hubs’ strength between each of MS subtype and HCs we found that patients exhibited reduced strength shown in a disease-specific pattern. For instance, in RRMS, a condition thought to have less tissue loss than SPMS, reduced strength was detected in a subset of hubs when compared to controls whereas in SPMS, which is the condition with a relatively high neurodegenerative component^28^ reduced strength was exhibited in the majority of hubs. A post-hoc analysis showed that reduced strength is associated with reduced brain volume (p = 0.034) which further supports our findings.

### Principal network organisation

PN decomposition is a novel technique that allowed us to disentangle the different subnetworks based on the brain’s internal connectivity^13^. The PN approach allows the researcher to delineate the networks into subnetworks in a non-mutually exclusive way^13^. For example, if a brain node is part of one PN, it can also be part of another PN if certain criteria are met. This is the case in biology in which it is known that a brain area can be part of more than one network i.e. the thalamus serves as a relay region in the visual and auditory pathways^29^. The application on HCs demonstrated that hub nodes form specific subnetworks (i.e. PN1 and PN2) and also there is high overlap of nodes in both subnetworks as assessed qualitatively.

On clinical-radiological grounds (lesion load and EDSS), the progressive MS subtypes (PPMS/SPMS) exhibit greater disease burden than the RRMS group although a few RRMS patients have high EDSS due to accrual of disability. The SPMS group however showed network differences not apparent in the PPMS group despite similar EDSS and lesion load. The SPMS group have reduced PN1/2 size **(**Fig. 1**)** and show more nodes with reduced strength than PPMS compared to HCs **(**Table 2**)**. This suggests that there might be subtle differences between SPMS and PPMS that manifest after network analysis, but this requires further study with larger cohort numbers. Additionally, the right thalamus was not included in SPMS. This is not surprising as there is reduced thalamic strength in SPMS compared to HCs **(**Table 2**)**. Additionally, a further post-hoc analysis showed that smaller deep grey matter atrophy is associated with reduced strength of subnetwork nuclei after controlling for age, gender, lesion load and total intracranial volume suggesting that tissue atrophy relates to reduced connections towards those deep nuclei atrophy. The findings from the hub approach, looking at the strength of individual nodes, and PNs approach, studying the hub connections among each other, are mutually supportive and further strengthened by this post-hoc analysis. Also, impaired thalamocortical connections are reported elsewhere^30^ supporting our findings and suggesting that our technique is sensitive to such changes. Although several hypothesis have been proposed to explain this loss^31^, the exact mechanisms are yet to be elucidated.

The study findings show that the right deep grey matter regions are involved in MS subtypes. Recent studies have shown asymmetrical damage accumulation^32–34^. Hand dominance has been suggested to be associated with higher lesion in the dominant hemisphere^35^. Unfortunately, due to the lack of such data we could not assess this further.

Other brain organisation features that have been studied in neurological disorders including MS, include the rich-club organisation and modularity. The former is the tendency of hub nodes to be more interconnected amongst themselves^10^. Disrupted rich-club organisation was reported in Alzheimer’s disease^36^ while decreased strength within the rich-club was reported in clinically isolated syndrome (CIS)^11^ and in PPMS^12^. Recently, it has been demonstrated that there are nodes common to PNs and to rich-club organisation^37^, highlighting that there is a subset of nodes with specific role in the network independent of the choice of the decomposition method. Modularity describes how well a network is divided into modules, which are nodes that are densely interconnected and have sparse connections to other modules. Network modularity has been recently investigated in several functional and structural MS network studies attesting to its clinical relevance^5,9,38^. Mapping out structural linkages of nodes and how these are affected in pathology could serve as a framework to identify subtle subnetwork changes.

### Clinical relevance of principal networks

The PNs are relevant to disability. Previous studies have reported that loss of thalamus volume increases the risk of EDSS worsening during follow-up^28^. Here, we showed that the connections of the subnetworks of the thalamus are associated with EDSS beyond volume of deep nuclei. Similarly, putamen plays a crucial role in MS as putamen atrophy starts directly after initial symptom manifestation or even years before and it correlates with EDSS^39^. In our study, we extended these findings showing that subnetwork putamen strength is associated with motor disability beyond deep grey matter volume measures. Thus, our results highlight the importance of connection integrity of specific deep nuclei in physical disability independently of tissue loss.

The PNs are also relevant to information processing speed performance. The importance of deep grey matter volume in cognition is shown elsewhere^8,40^. The findings presented here highlight that intact connections of the second PN thalamus and putamen are important in maintaining information processing speed functioning beyond participant’s tissue volume. Pathological features of impaired connections include neuroaxonal damage, inflammation and neurodegeneration^41^. The association between thalamus strength of PN1 was only marginally associated with SDMT suggesting that maybe for cognition the PN2 is more sensitive in capturing such changes. Future studies could address this.

### Limitations and future direction

There is no consensus as to how network hubs are defined^23^. Here, we used strength which has been used before in MS^4^. Additionally, the derived PNs do not correspond to previous work^13^. However, this could be due to differences in the tractogram reconstruction in which we used CSD, ACT and SIFT2 which alter internal connectivity and hence influence the derived subnetworks which are heavily dependent on the underlying connectivity. The effect of tractogram reconstruction techniques and PN analysis is beyond the scope of this study. Moreover, in this study we used the latest techniques to address reconstruction biases as shown previously^8^. However, histological validations are imperative to make direct links between imaging and pathology. The reduced number of participants with SDMT scores is discussed elsewhere^8^. Finally, we did not perform multiple comparisons correction. However, this is an exploratory study and we therefore believe that it is not appropriate to risk missing any significant associations due to conservative post-hoc analyses. Future studies could confirm the findings presented here.

Future studies could also assess if hubs are still connected to the same nodes in MS or if their connectivity changes. Additionally, hubs and their connections might be particularly vulnerable to pathogenic factors due their central role in the network^42^, therefore future work could study whether there is a higher probability of lesions being present in PNs and/or whether the presence of lesions in the PNs is more detrimental for accumulation of disability. Additionally, both putamen and thalamus have shown the strongest volume changes upon treatment^43^ and hence it will be interesting to investigate how treatment affects PNs and if such changes are clinically relevant. Previous studies have suggested that the first two PNs typically capture the major connectivity features of the network, but the threshold is ultimately arbitrary. Investigation of more PNs could be addressed in future studies. Finally, longitudinal studies could investigate how the patterns of connectivity change over time and if they change in the same way in all MS subtypes.

## Conclusion

In conclusion, we report that network hubs are relatively consistent across the various MS subtypes and in HCs. Additionally, a subset of network hubs forms specific PNs and specific nodal strengths within these subnetworks are associated with clinical disability and information processing speed dysfunction. These results highlight the potential utility of subnetwork-based approaches as imaging biomarkers for disease progression and for assessing treatment effects.

## Supplementary information

Supplementary information.
