# Supplementary material for: Disrupted principal network organisation in multiple sclerosis relates to disability
Source: Sci Rep. 2020 Feb 27;10:3620. doi: 10.1038/s41598-020-60611-4 (PMC7046772; doi:10.1038/s41598-020-60611-4)
Supplement: Supplementary file 1 — Supplementary information. [file 41598_2020_60611_MOESM1_ESM.pdf]

Disrupted principal network organisation in multiple sclerosis relates to disability

Thalis Charalambous[1], Jonathan D. Clayden[2], Elizabeth Powell[1,3], Ferran Prados[1,4,5], Carmen Tur[1], Baris Kanber[1,4], Declan Chard[1], Sebastien Ourselin[4], Claudia A.M. Gandini Wheeler-Kingshott[1,6,7], Alan J. Thompson[1], Ahmed T. Toosy[1]

[1] Queen Square MS Centre, Department of Neuroinflammation, UCL Institute of Neurology, Faculty of Brain Sciences, University College London

[2] UCL GOS Institute of Child Health, University College London, London, UK

[3] Medical Physics and Biomedical Engineering, University College London, London, UK

[4] Center for Medical Imaging Computing, Medical Physics and Biomedical Engineering, UCL, London WC1V 6LJ, UK

[5] eHealth Centre, Universitat Oberta de Catalunya, Barcelona, Spain

[6] Brain MRI 3T Research Center, C. Mondino National Neurological Institute, Pavia, Italy

[7] Department of Brain and Behavioural Sciences, University of Pavia, Pavia, Italy

eTable 1: Characteristics of the participants with and without SDMT score

	Whole MS population (n = 122)	MS Population with SDMT (n = 60)	MS Population without SDMT (n = 62)	p value
**Subtypes**	RRMS = 58<br>PPMS = 28<br>SPMS = 36	RRMS = 28<br>PPMS = 14<br>SPMS = 18	RRMS = 29<br>PPMS = 15<br>SPMS = 18	0.98[a]
**Age, years**	48 ± 11	47 ± 11	49 ± 10	0.48[b]
**Gender (M/F)**	36/86	18/42	18/44	0.99[a]
**Disease duration, years**	15 ± 10	16 ± 11	14 ± 8	0.29[b]
**EDSS, median**	5.5 (0-8.5)	4.5 (1.0-8.5)	6 (0 - 8.5)	0.07[b]

Values listed are mean ± standard deviation (SD)

Comparison was performed between the MS population with and without SDMT

[a] chi Square test

[b] Student t-test for independent samples

Abbreviations: MS = multiple sclerosis; RRMS = relapsing-remitting MS; PPMS = primary progressive MS; SPMS = secondary progressive MS; SDMT = Symbol Digit Modality Test; EDSS = Expanded Disability Scale Status

eFigure1: The first principal network in controls and MS subtypes in (A) side view and (B) front view

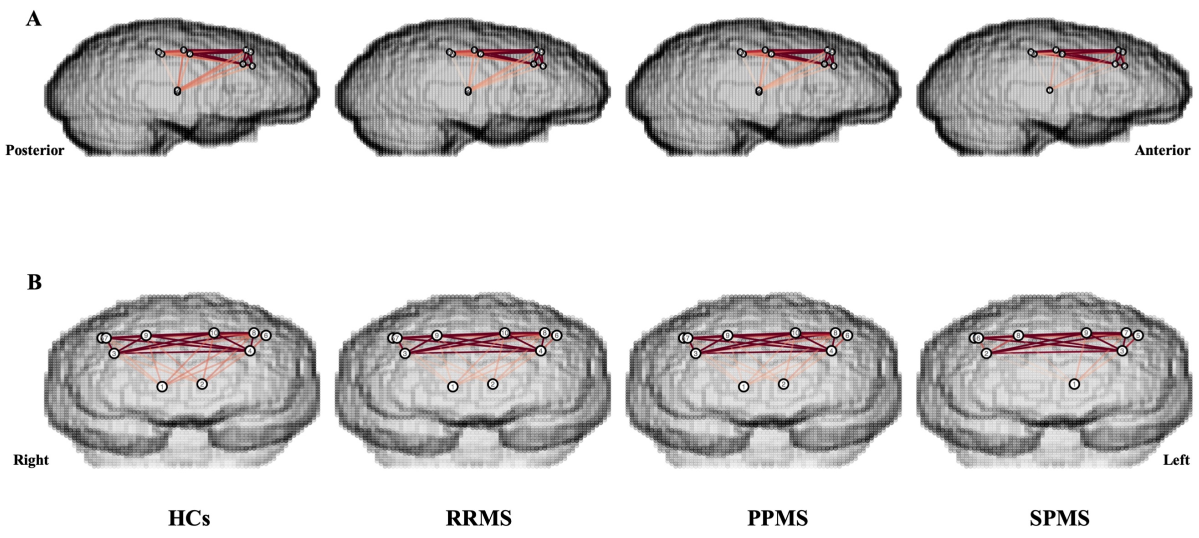

eFigure2: The second principal network in controls and MS subtypes in (A) side view and (B) front view

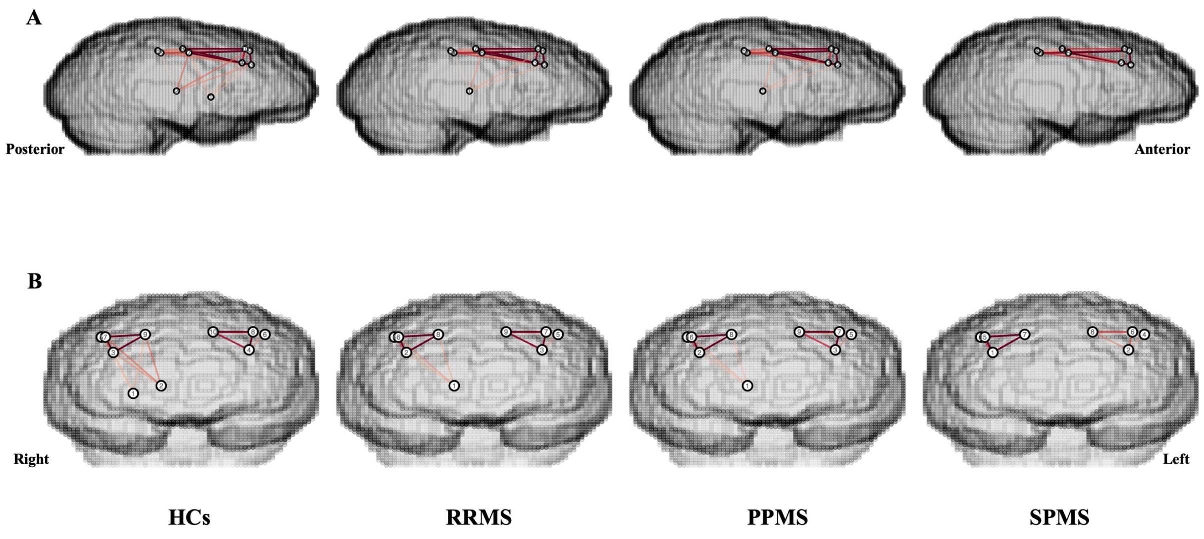

Supplemental results

We have constructed a pairwise univariate association matrix that includes all brain regions belonging to the PN1 and PN2 **(eFigure 3)**. This exploratory analysis shows that the higher correlation coefficient (r) between clinical disability and brain regions is shown in PN1 right thalamus, PN2 right thalamus and PN2 and right putamen. More specifically, high EDSS score is associated with reduced PN1 thalamus strength (r=-0.3), reduced PN2 right putamen strength (r=-0.4) and reduced PN2 right thalamus strength (r=-0.3). The correlation coefficient of the other regions is below -0.3. For SDMT, we showed that low SDMT correlates with reduced PN1 thalamus strength (r=0.3), reduced PN2 right putamen strength (r=0.6) and reduced PN2 right thalamus strength (r=0.5). The other region that showed some association with SDMT is PN1 left thalamus which demonstrated that low SDMT values correlated with reduced strength of these regions (r=0.3). A post-hoc analysis, showed that there was no significant association between PN1 left thalamus with SDMT score after adjusting for age, gender and lesion load.

We did not perform regression analyses between the other brain regions belong to PN1 and PN2 with clinical disability because there were very weak correlation between these variables (r is between -0.2 to 0.2) and hence it is unlikely to give any significant association.

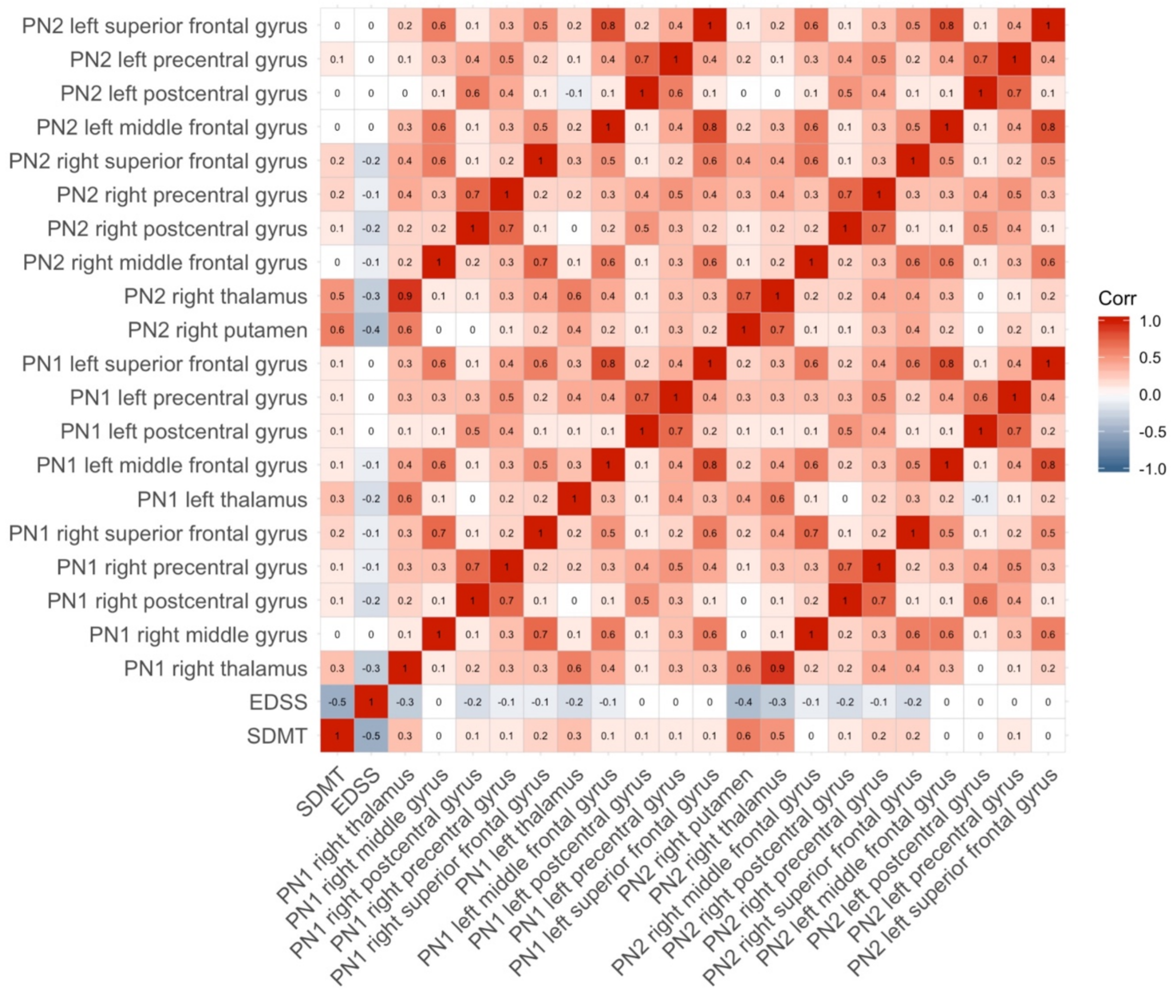

eFigure 3. Exploratory pairwise univariate associations.

The value in each element corresponds to the pairwise Pearson correlation coefficient (r).

Abbreviations

PN = principal network; EDSS = Expanded Disability Status Scale; SDMT = Symbol Digit Modalities Test
